# Woman’s Temperance Convention

**Published:** 1875-11

**Authors:** 


### WOMAN'S TEMPERANCE CONVENTION.

The Woman's National Temperance Convention recently held a spirited session in Cincinnati. They adopted resolutions favoring temperance prayer-meetings, cheap lodging-houses, and free reading-rooms for inebriates. They also recommended each State to memorialize Congress to appoint a national committee of medical men to report upon the injury done by the traffic in alcoholic drinks. It is evident that the women are in earnest in their temperance work, and that the scourge of intemperance is receiving severe blows from those who feel most keenly the need of reform, and who suffer most from the ravages of the whiskey pestilence. Perhaps we shall never have genuine and inflexible legislation against alcoholic traffic until the ballot is in the hands of deserving woman. That her voice and vote will be in favor of a full suppression of the traffic, instead of half-way measures, we feel sure; and for this, if for no other reason, we shall rejoice when the power is given her to dethrone the monarch which has brought so much misery to her especial sanctuary—the home.

---

NOTHING is intolerable that is necessary.

